# Poly[[aqua­[μ_3_-(2,6-^2^H_2_)-isonicotinato-κ^3^ *N*:*O*:*O*′][μ_2_-(2,6-^2^H_2_)-isonicotinato-κ^2^ *N*:*O*]manganese(II)] ethanol solvate]

**DOI:** 10.1107/S1600536808016206

**Published:** 2008-07-16

**Authors:** Wei Dai

**Affiliations:** a Ordered Matter Science Research Center, College of Chemistry and Chemical Engineering, Southeast University, Nanjing 210096, People’s Republic of China

## Abstract

In the title compound, {[Mn(C_6_H_2_D_2_NO_2_)_2_(H_2_O)]·C_2_H_6_O}_*n*_, the Mn^II^ metal centre displays a slightly distorted octa­hedral coordination geometry, provided by three O and two N atoms of five isonicotinate ligands and one O atom of a water mol­ecule. There are two types of isonicotinate anions, one acting as a bridging tridentate group and the other in a bridging bidentate fashion, to form a polymeric three-dimensional network. The structure is stabilized by intra- and inter­molecular O—H⋯O and C—H⋯O hydrogen-bond inter­actions.

## Related literature

For related literature, see: Akutagawa *et al.* (2004 ▶); Cova *et al.* (2001 ▶); Pavlik & Laohhasurayotin (2005 ▶); Sekiya & Nishikiori (2001 ▶).
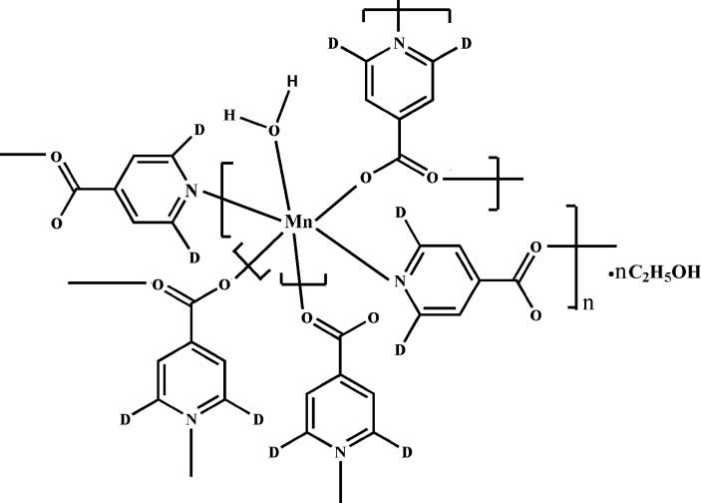


## Experimental

### 

#### Crystal data

[Mn(C_6_H_2_D_2_NO_2_)_2_(H_2_O)]·C_2_H_6_O
*M* *_r_* = 367.24
Monoclinic, 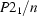

*a* = 10.903 (2) Å
*b* = 12.180 (2) Å
*c* = 13.015 (3) Å
β = 110.02 (3)°
*V* = 1623.9 (6) Å^3^
*Z* = 4
Mo *K*α radiation
μ = 0.84 mm^−1^
*T* = 293 (2) K
0.20 × 0.20 × 0.20 mm

#### Data collection

Rigaku Mercury2 diffractometer
Absorption correction: multi-scan (*CrystalClear*; Rigaku, 2005 ▶) *T*_min_ = 0.795, *T*_max_ = 0.841
16339 measured reflections
3701 independent reflections
3087 reflections with *I* > 2σ(*I*)
*R*_int_ = 0.042

#### Refinement

*R*[*F* ^2^ > 2σ(*F* ^2^)] = 0.040
*wR*(*F* ^2^) = 0.142
*S* = 1.06
3701 reflections
248 parameters
H atoms treated by a mixture of independent and constrained refinement
Δρ_max_ = 0.52 e Å^−3^
Δρ_min_ = −0.52 e Å^−3^

### 

Data collection: *CrystalClear* (Rigaku, 2005 ▶); cell refinement: *CrystalClear*; data reduction: *CrystalClear*; program(s) used to solve structure: *SHELXS97* (Sheldrick, 2008 ▶); program(s) used to refine structure: *SHELXL97* (Sheldrick, 2008 ▶); molecular graphics: *SHELXTL* (Sheldrick, 2008 ▶); software used to prepare material for publication: *SHELXL97*.

## Supplementary Material

Crystal structure: contains datablocks I, global. DOI: 10.1107/S1600536808016206/rz2216sup1.cif

Structure factors: contains datablocks I. DOI: 10.1107/S1600536808016206/rz2216Isup2.hkl

Additional supplementary materials:  crystallographic information; 3D view; checkCIF report

## Figures and Tables

**Table 1:** Hydrogen-bond geometry (Å, °)

*D*—H⋯*A*	*D*—H	H⋯*A*	*D*⋯*A*	*D*—H⋯*A*
C11—H11*A*⋯O5	0.98 (4)	2.44 (3)	2.784 (3)	100 (2)
O4—H2*W*⋯O6	0.79 (4)	1.90 (4)	2.680 (5)	166 (4)
O6—H6*A*⋯O3^i^	0.85	1.90	2.729 (3)	165
C11—H11*A*⋯O3^ii^	0.98 (4)	2.50 (4)	3.404 (4)	153 (3)
O4—H1*W*⋯O3^ii^	0.92 (4)	1.89 (4)	2.793 (3)	166 (3)

Symmetry codes: (i) 
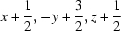
; (ii) 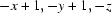.

**Fig. 1.**
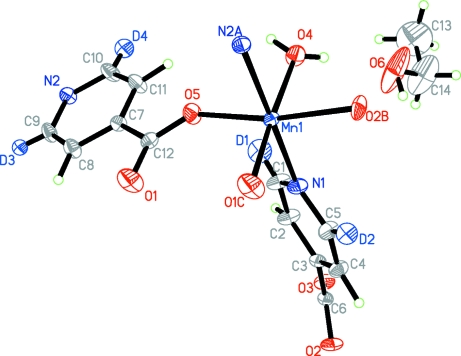
A view of the title compound showing the coordination around the manganese(II) atom. Displacement ellipsoids are drawn at the 30% probability level. [Symmetry codes: (A) 1/2+x, 1/2-y, 1/2+z; (B) 1/2+x, 3/2-y; (C) 1/2+z and 2-x, 1-y, -z]

**Fig. 2.**
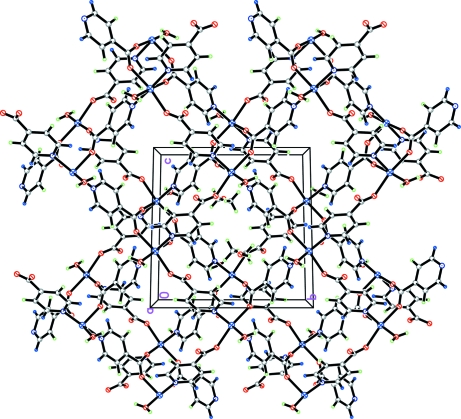
Packing diagram of the title compound viewed along the a axis. Ethanol solvent molecules are omitted for clarity.

## supplementary crystallographic information

### Comment

Isonicotinic acid is a good mono- or bidentate ligand for the construction of supramolecular complexes with versatile binding modes (Cova *et al.*, 2001; Sekiya & Nishikiori, 2001). Until now, a large number of metal-organic framework structures containing isonicotinic acid ligands have been reported. Investigations on the effect of deuteration onto the physical properties like permittivity has become of increasing interest (Akutagawa *et al.*, 2004).

In the crystal structure of the title compound the manganese atom displays a slightly distorted octahedral geometry provided by two N atoms and three carboxylate O atoms of five different isonicotinato anions and one O atom of a water molecule (Fig. 1). The structure contains two types of isonicotinato ligands, one acting as a bridging trichelate group, the other as bridging bidentate group to form a polymeric three-dimensional network (Fig. 2). The shortest interatomic Mn···Mn separation is 4.9182 (12) Å. The structure is stabilized by intra- and intermolecular O—H···O and C—H···O hydrogen bonds (Table 1).

### Experimental

A mixture of isonicotinic acid N-oxide (21.6 mmol) in deuterium oxide (10.0 mL) and sodium deuteroxide (31.0 mmol) was acidified with concentrated hydrochloric acid. After 4 h isonicotinic acid N-oxide-2,6-D_2_ was separated as a white solid. A solution of this compound (9.0 mmol) in dichloromethane (60 mL) was added dropwise to phosphorus trichloride (1.2 mL). The mixture was refluxed for 1 h, mixed with ice–water (30 mL), made alkaline with aqueous NaOH (10 N) and extracted with dichloromethane (5 x 20mL) according to the method reported by Pavlik & Laohhasurayotin (2005). The organic phase was dried over anhydrous sodium sulfate to  give isonicotinic acid-2,6-D_2_. A mixture of isonicotinic acid-2,6-D_2_ (0.1 mmol), manganese(II) acetate (0.2 mmol), ethanol (1 ml) and water (0.1 ml) was then transferred into a sealed Pyrex tube and heated at 100°C for 2 d. Yellow crystals of the title compound suitable for X-ray analysis were obtained on slow cooling to room temperature.

### Refinement

H and D atoms associated with the pyridine rings and water molecule were located in a difference Fourier map and refined freely. All other H atoms were placed in calculated positions, with C—H = 0.86–0.96 Å, O—H = 0.85 Å, and with *U*_iso_(H) = 1.5 *U*_eq_(C, O) or 1.5 *U*_eq_(C) for methylene H atoms.

### Crystal data

**Table:**

[Mn(C_6_H_2_D_2_NO_2_)_2_(H_2_O)]·C_2_H_6_O	*F*_000_ = 748
*M**_r_* = 367.24	*D*_x_ = 1.502 Mg m^−^^3^
Monoclinic, *P*2_1_/*n*	Mo *K*α radiation λ = 0.71073 Å
Hall symbol: -P 2yn	Cell parameters from 19580 reflections
*a* = 10.903 (2) Å	θ = 3.0–27.5º
*b* = 12.180 (2) Å	µ = 0.84 mm^−^^1^
*c* = 13.015 (3) Å	*T* = 293 (2) K
β = 110.02 (3)º	Block, yellow
*V* = 1623.9 (6) Å^3^	0.20 × 0.20 × 0.20 mm
*Z* = 4	

### Data collection

**Table:**

Rigaku Mercury2 (2x2 bin mode) diffractometer	3701 independent reflections
Radiation source: fine-focus sealed tube	3087 reflections with *I* > 2σ(*I*)
Monochromator: graphite	*R*_int_ = 0.042
Detector resolution: 13.6612 pixels mm^-1^	θ_max_ = 27.5º
*T* = 293(2) K	θ_min_ = 3.0º
CCD profile fitting scans	*h* = −14→14
Absorption correction: multi-scan(CrystalClear; Rigaku, 2005)	*k* = −15→15
*T*_min_ = 0.795, *T*_max_ = 0.841	*l* = −16→16
16339 measured reflections	

### Refinement

**Table:**

Refinement on *F*^2^	Secondary atom site location: difference Fourier map
Least-squares matrix: full	Hydrogen site location: inferred from neighbouring sites
*R*[*F*^2^ > 2σ(*F*^2^)] = 0.040	H atoms treated by a mixture of independent and constrained refinement
*wR*(*F*^2^) = 0.142	*w* = 1/[σ^2^(*F*_o_^2^) + (0.1*P*)^2^] where *P* = (*F*_o_^2^ + 2*F*_c_^2^)/3
*S* = 1.06	(Δ/σ)_max_ < 0.001
3701 reflections	Δρ_max_ = 0.52 e Å^−^^3^
248 parameters	Δρ_min_ = −0.52 e Å^−^^3^
Primary atom site location: structure-invariant direct methods	Extinction correction: none

### Special details

**Table:**

Geometry. All e.s.d.'s (except the e.s.d. in the dihedral angle between two l.s. planes) are estimated using the full covariance matrix. The cell e.s.d.'s are taken into account individually in the estimation of e.s.d.'s in distances, angles and torsion angles; correlations between e.s.d.'s in cell parameters are only used when they are defined by crystal symmetry. An approximate (isotropic) treatment of cell e.s.d.'s is used for estimating e.s.d.'s involving l.s. planes.
Refinement. Refinement of *F*^2^ against ALL reflections. The weighted *R*-factor *wR* and goodness of fit *S* are based on *F*^2^, conventional *R*-factors *R* are based on *F*, with *F* set to zero for negative *F*^2^. The threshold expression of *F*^2^ > σ(*F*^2^) is used only for calculating *R*-factors(gt) *etc*. and is not relevant to the choice of reflections for refinement. *R*-factors based on *F*^2^ are statistically about twice as large as those based on *F*, and *R*- factors based on ALL data will be even larger.

### Fractional atomic coordinates and isotropic or equivalent isotropic displacement parameters (Å^2^)

**Table:**

	*x*	*y*	*z*	*U*_iso_*/*U*_eq_	
Mn1	0.93671 (3)	0.50175 (2)	0.16410 (2)	0.02182 (15)	
O5	0.87201 (17)	0.38008 (13)	0.03681 (14)	0.0391 (4)	
O4	0.79806 (18)	0.41661 (16)	0.22877 (17)	0.0411 (4)	
N1	0.77113 (19)	0.61593 (15)	0.06381 (16)	0.0334 (4)	
C7	0.7734 (2)	0.27522 (17)	−0.12181 (17)	0.0260 (4)	
C12	0.8682 (2)	0.36318 (16)	−0.05830 (18)	0.0268 (4)	
C11	0.6930 (3)	0.2208 (2)	−0.0751 (2)	0.0395 (6)	
C5	0.7818 (2)	0.72543 (19)	0.0584 (2)	0.0376 (5)	
C8	0.7617 (2)	0.2480 (2)	−0.2283 (2)	0.0353 (5)	
C1	0.6582 (2)	0.5717 (2)	0.0011 (2)	0.0404 (6)	
C2	0.5536 (2)	0.63237 (19)	−0.0664 (2)	0.0395 (6)	
C3	0.5668 (2)	0.74530 (17)	−0.07336 (16)	0.0273 (4)	
C4	0.6847 (2)	0.79184 (19)	−0.00909 (19)	0.0358 (5)	
C6	0.4577 (2)	0.81308 (17)	−0.15183 (17)	0.0274 (4)	
O3	0.34326 (15)	0.77890 (14)	−0.17448 (14)	0.0386 (4)	
O2	0.49329 (16)	0.89829 (13)	−0.18801 (14)	0.0385 (4)	
O1	0.9325 (2)	0.40991 (17)	−0.10687 (17)	0.0554 (5)	
C10	0.6056 (3)	0.1433 (2)	−0.1362 (2)	0.0409 (6)	
C9	0.6720 (2)	0.1688 (2)	−0.2837 (2)	0.0355 (5)	
N2	0.59483 (18)	0.11618 (15)	−0.23923 (15)	0.0311 (4)	
O6	0.6716 (3)	0.5555 (2)	0.3174 (4)	0.1366 (18)	
H6A	0.7151 (3)	0.6101 (2)	0.3078 (4)	0.205*	
C14	0.5578 (4)	0.5893 (4)	0.3214 (6)	0.118 (2)	
H14A	0.5176 (4)	0.6222 (4)	0.2618 (6)	0.142*	
H14B	0.5716 (4)	0.6358 (4)	0.3729 (6)	0.142*	
C13	0.4751 (7)	0.5076 (5)	0.3359 (7)	0.132 (3)	
H13A	0.3950 (7)	0.5394 (5)	0.3370 (7)	0.197*	
H13B	0.4566 (7)	0.4556 (5)	0.2771 (7)	0.197*	
H13C	0.5183 (7)	0.4711 (5)	0.4041 (7)	0.197*	
H8A	0.804 (3)	0.287 (2)	−0.269 (2)	0.038 (7)*	
D3	0.662 (2)	0.1472 (19)	−0.355 (2)	0.028 (6)*	
D1	0.648 (4)	0.495 (3)	0.008 (3)	0.063 (12)*	
D4	0.549 (3)	0.109 (2)	−0.105 (3)	0.044 (7)*	
H4A	0.701 (3)	0.872 (2)	−0.015 (2)	0.044 (7)*	
D2	0.864 (3)	0.758 (2)	0.109 (2)	0.044 (7)*	
H11A	0.708 (3)	0.236 (3)	0.002 (3)	0.065 (10)*	
H2A	0.472 (3)	0.591 (3)	−0.105 (3)	0.066 (10)*	
H1W	0.758 (4)	0.351 (3)	0.202 (3)	0.069 (10)*	
H2W	0.751 (4)	0.450 (3)	0.252 (3)	0.066 (11)*	

### Atomic displacement parameters (Å^2^)

**Table:**

	*U*^11^	*U*^22^	*U*^33^	*U*^12^	*U*^13^	*U*^23^
Mn1	0.0206 (2)	0.0197 (2)	0.0215 (2)	−0.00130 (10)	0.00247 (15)	−0.00019 (10)
O5	0.0464 (10)	0.0376 (9)	0.0314 (9)	−0.0144 (7)	0.0108 (7)	−0.0118 (7)
O4	0.0420 (10)	0.0345 (9)	0.0554 (11)	−0.0090 (8)	0.0275 (9)	−0.0037 (8)
N1	0.0309 (10)	0.0305 (9)	0.0331 (10)	0.0022 (8)	0.0037 (8)	0.0083 (7)
C7	0.0240 (10)	0.0263 (10)	0.0262 (10)	−0.0052 (8)	0.0067 (8)	−0.0016 (8)
C12	0.0233 (10)	0.0227 (10)	0.0313 (11)	−0.0033 (8)	0.0054 (8)	−0.0014 (8)
C11	0.0475 (14)	0.0470 (13)	0.0266 (12)	−0.0224 (11)	0.0159 (10)	−0.0072 (10)
C5	0.0368 (13)	0.0316 (11)	0.0339 (12)	−0.0019 (10)	−0.0014 (10)	0.0003 (9)
C8	0.0331 (12)	0.0427 (14)	0.0344 (12)	−0.0145 (10)	0.0173 (10)	−0.0070 (10)
C1	0.0309 (12)	0.0290 (12)	0.0532 (15)	−0.0014 (9)	0.0042 (10)	0.0125 (10)
C2	0.0299 (12)	0.0307 (12)	0.0488 (15)	−0.0029 (9)	0.0018 (10)	0.0105 (10)
C3	0.0280 (10)	0.0297 (11)	0.0237 (10)	0.0024 (8)	0.0083 (8)	0.0076 (8)
C4	0.0389 (13)	0.0268 (11)	0.0337 (12)	−0.0038 (10)	0.0022 (10)	0.0015 (9)
C6	0.0308 (11)	0.0272 (10)	0.0228 (10)	0.0021 (8)	0.0074 (8)	0.0046 (8)
O3	0.0279 (8)	0.0365 (9)	0.0472 (10)	0.0006 (7)	0.0074 (7)	0.0112 (7)
O2	0.0381 (9)	0.0329 (8)	0.0362 (9)	−0.0031 (7)	0.0021 (7)	0.0171 (7)
O1	0.0586 (12)	0.0619 (12)	0.0511 (12)	−0.0404 (10)	0.0255 (10)	−0.0110 (9)
C10	0.0474 (14)	0.0483 (14)	0.0303 (12)	−0.0261 (12)	0.0175 (11)	−0.0060 (10)
C9	0.0390 (13)	0.0431 (13)	0.0277 (12)	−0.0136 (10)	0.0157 (10)	−0.0124 (9)
N2	0.0320 (10)	0.0329 (10)	0.0261 (9)	−0.0122 (8)	0.0071 (7)	−0.0055 (7)
O6	0.103 (2)	0.0659 (18)	0.295 (5)	−0.0334 (17)	0.138 (3)	−0.077 (3)
C14	0.064 (3)	0.085 (3)	0.216 (7)	−0.019 (2)	0.062 (3)	−0.044 (3)
C13	0.098 (5)	0.143 (6)	0.184 (8)	−0.016 (3)	0.087 (5)	0.011 (4)

### Geometric parameters (Å, °)

**Table:**

Mn1—O1^i^	2.1151 (18)	C2—H2A	1.00 (3)
Mn1—O5	2.1537 (16)	C2—C3	1.389 (3)
Mn1—O2^ii^	2.1806 (16)	C3—C4	1.392 (3)
Mn1—O4	2.2228 (18)	C3—C6	1.519 (3)
Mn1—N2^iii^	2.2656 (18)	C4—H4A	1.00 (3)
Mn1—N1	2.2987 (19)	C6—O3	1.251 (3)
O5—C12	1.242 (3)	C6—O2	1.255 (3)
O4—H2W	0.79 (4)	O2—Mn1^iv^	2.1806 (16)
O4—H1W	0.92 (4)	O1—Mn1^i^	2.1151 (18)
N1—C1	1.336 (3)	C10—N2	1.346 (3)
N1—C5	1.343 (3)	C10—D4	0.95 (3)
C7—C8	1.388 (3)	C9—N2	1.336 (3)
C7—C11	1.393 (3)	C9—D3	0.93 (3)
C7—C12	1.521 (3)	N2—Mn1^v^	2.2656 (18)
C12—O1	1.232 (3)	O6—H6A	0.8499
C11—H11A	0.98 (4)	O6—C14	1.325 (5)
C11—C10	1.384 (3)	C14—H14A	0.8499
C5—C4	1.382 (3)	C14—H14B	0.8500
C5—D2	0.99 (3)	C14—C13	1.397 (7)
C8—H8A	0.95 (3)	C13—H13A	0.9599
C8—C9	1.387 (3)	C13—H13C	0.9600
C1—C2	1.390 (3)	C13—H13B	0.9602
C1—D1	0.94 (3)		
			
O1^i^—Mn1—O5	99.32 (7)	N1—C1—D1	117 (2)
O1^i^—Mn1—O2^ii^	90.33 (8)	C2—C1—D1	119 (2)
O5—Mn1—O2^ii^	169.24 (7)	H2A—C2—C3	124.0 (19)
O1^i^—Mn1—O4	177.07 (8)	H2A—C2—C1	117 (2)
O5—Mn1—O4	83.34 (7)	C3—C2—C1	119.1 (2)
O2^ii^—Mn1—O4	87.12 (8)	C2—C3—C4	117.3 (2)
O1^i^—Mn1—N2^iii^	92.38 (8)	C2—C3—C6	120.46 (19)
O5—Mn1—N2^iii^	88.71 (7)	C4—C3—C6	122.16 (19)
O2^ii^—Mn1—N2^iii^	86.11 (7)	H4A—C4—C5	120.1 (17)
O4—Mn1—N2^iii^	88.90 (7)	H4A—C4—C3	120.4 (17)
O1^i^—Mn1—N1	89.17 (8)	C5—C4—C3	119.5 (2)
O5—Mn1—N1	89.58 (7)	O3—C6—O2	126.75 (19)
O2^ii^—Mn1—N1	95.35 (7)	O3—C6—C3	117.77 (18)
O4—Mn1—N1	89.63 (7)	O2—C6—C3	115.47 (18)
N2^iii^—Mn1—N1	177.87 (6)	C6—O2—Mn1^iv^	139.70 (15)
C12—O5—Mn1	140.47 (14)	C12—O1—Mn1^i^	170.48 (18)
H2W—O4—H1W	107 (3)	N2—C10—C11	123.2 (2)
H2W—O4—Mn1	122 (3)	N2—C10—D4	118.4 (18)
H1W—O4—Mn1	124 (2)	C11—C10—D4	118.4 (18)
C1—N1—C5	116.52 (19)	N2—C9—C8	123.0 (2)
C1—N1—Mn1	118.92 (15)	N2—C9—D3	114.5 (15)
C5—N1—Mn1	124.44 (15)	C8—C9—D3	122.4 (15)
C8—C7—C11	117.73 (19)	C9—N2—C10	117.39 (19)
C8—C7—C12	121.61 (18)	C9—N2—Mn1^v^	122.39 (15)
C11—C7—C12	120.64 (19)	C10—N2—Mn1^v^	119.92 (14)
O1—C12—O5	127.2 (2)	H6A—O6—C14	110
O1—C12—C7	116.5 (2)	H14A—C14—H14B	107.5
O5—C12—C7	116.30 (18)	H14A—C14—O6	108
H11A—C11—C10	124 (2)	H14B—C14—O6	109
H11A—C11—C7	117 (2)	H14A—C14—C13	108
C10—C11—C7	119.1 (2)	H14B—C14—C13	108
N1—C5—C4	123.6 (2)	O6—C14—C13	116.1 (5)
N1—C5—D2	115.9 (16)	H13A—C13—H13C	109.5
C4—C5—D2	120.4 (16)	H13A—C13—H13B	109.5
H8A—C8—C9	116.9 (16)	H13C—C13—H13B	109.5
H8A—C8—C7	123.2 (16)	H13A—C13—C14	110
C9—C8—C7	119.5 (2)	H13C—C13—C14	109
N1—C1—C2	123.9 (2)	H13B—C13—C14	109

Symmetry codes: (i) −*x*+2, −*y*+1, −*z*; (ii) *x*+1/2, −*y*+3/2, *z*+1/2; (iii) *x*+1/2, −*y*+1/2, *z*+1/2; (iv) *x*−1/2, −*y*+3/2, *z*−1/2; (v) *x*−1/2, −*y*+1/2, *z*−1/2.

### Hydrogen-bond geometry (Å, °)

**Table:**

*D*—H···*A*	*D*—H	H···*A*	*D*···*A*	*D*—H···*A*
C11—H11A···O5	0.98 (4)	2.44 (3)	2.784 (3)	100 (2)
O4—H2W···O6	0.79 (4)	1.90 (4)	2.680 (5)	166 (4)
O6—H6A···O3^ii^	0.85	1.90	2.729 (3)	165
C11—H11A···O3^vi^	0.98 (4)	2.50 (4)	3.404 (4)	153 (3)
O4—H1W···O3^vi^	0.92 (4)	1.89 (4)	2.793 (3)	166 (3)

Symmetry codes: (ii) *x*+1/2, −*y*+3/2, *z*+1/2; (vi) −*x*+1, −*y*+1, −*z*.
